# Profiling immunogenic neoantigen peptides elicited by personalized neoantigen vaccine in cancer patients

**DOI:** 10.3389/fimmu.2026.1829509

**Published:** 2026-06-09

**Authors:** Peng Zhao, Clara Effenberger, Saki Matsumoto, Takafumi Morisaki, Yu Ishii, Masayo Umebayashi, Hiroto Tanaka, Norihiro Koya, Shinichiro Nakagawa, Kenta Tsujimura, Yusuke Nakamura, Takashi Morisaki, Kazuma Kiyotani

**Affiliations:** 1 Laboratory of Immunogenomics, Center for Intractable Diseases and ImmunoGenomics (CiDIG), National Institutes of Biomedical Innovation, Health and Nutrition (NIBN), Ibaraki, Osaka, Japan; 2 Project for Immunogenomics, Cancer Precision Medicine Center, Japanese Foundation for Cancer Research, Tokyo, Japan; 3 Department of Cancer Immunotherapy, Fukuoka General Cancer Clinic, Fukuoka, Japan

**Keywords:** cancer immunotherapy, cancer vaccine, immunogenicity, neoantigen, T cell response

## Abstract

**Introduction:**

Personalized neoantigen vaccines can induce antitumor T cell responses, but only 10-20% of selected peptides have induced immune responses in patients, underscoring the limitations of current prediction strategies.

**Methods:**

We analyzed a clinically annotated dataset from 352 cancer patients who received personalized neoantigen peptide-pulsed dendritic cell vaccines. We focused on 2,317 short peptides derived from single nucleotide variants for which post-vaccination T cell responses were evaluated by IFN-γ ELISPOT assay. Immunogenic neoantigen peptides were defined as those inducing a ≥2.0-fold increase in IFN-γ ELISPOT responses after vaccination. We systematically examined peptide intrinsic characteristics and physicochemical properties, as well as predicted scores related to antigen-processing machinery.

**Results and discussion:**

Immunogenicity was not associated with specific mutation positions or sequence patterns but was significantly correlated with higher hydrophobicity (*P* = 5.2 × 10^-4^). Among several predictive scores, peptides with higher binding affinity to HLA molecules (*P* = 0.0014 for NetMHC3, *P* = 0.028 for MHCflurry-affinity), higher binding stability (*P* = 0.043 for NetMHCstab) or better peptide presentation scores (*P* = 0.012 for mixmhcPred3, *P* = 0.0085 for MHCflurry-presentation) were significantly enriched among immunogenic neoantigen peptides. Composite models integrating peptide physicochemical features, particularly hydrophobicity, with prediction scores improved the area under the receiver operating characteristic curve and balanced accuracy compared with individual tools alone. Together, these findings highlight the multifactorial determinants of neoantigen immunogenicity and support the integration of complementary peptide features to refine neoantigen prioritization for personalized vaccines and T cell-based immunotherapies.

## Introduction

Cancer immunotherapy has transformed the therapeutic landscape by enabling a patient’s own immune systems to recognize and eliminate malignant cells. A central focus of this approach is cancer-specific neoantigens, peptides derived from mutated proteins and presented on human leukocyte antigen (HLA) molecules (1). Because neoantigens are absent from normal tissues, they represent highly tumor-specific targets with minimal risk of off-target effects. Early clinical trials of personalized neoantigen vaccines have demonstrated that neoantigen-targeted vaccination is feasible and capable of inducing cancer-specific T cell responses, with some studies reporting durable clinical benefit (2–4).

Despite these promising findings, accurate prediction of truly immunogenic neoantigens remains a major challenge. Current prioritization strategies rely on computational pipelines that integrate tumor-normal sequencing, HLA typing, and peptide-HLA binding affinity predictions (2, 3). However, immunogenicity is governed by multiple additional biological processes. Neoantigen generation and presentation involve a series of highly regulated steps including proteasomal degradation of mutant proteins into peptides, transport of these peptides into the endoplasmic reticulum via TAP, trimming and loading onto specific HLA molecules, and stable presentation on the cell surface (5, 6). Furthermore, effective T cell recognition also depends on the host immune context, including T cell repertoire, prior antigen exposure, and tumor microenvironmental factors. This multifactorial complexity likely explains why only a small fraction (<20-30%) of predicted neoantigen candidates elicit detectable T cell responses *in vivo* (2–4, 7).

To improve neoantigen vaccine design, it is essential to identify peptide-level and biological features that distinguish immunogenic neoantigens from non-immunogenic ones, and to determine whether integration of such features with current *in silico* pipelines can enhance the selection of clinically relevant targets. In this study, we analyzed a clinically annotated dataset of 2,317 neoantigen peptides used for personalized neoantigen peptide-pulsed dendritic cell (DC) vaccines in 352 cancer patients. Through systematic evaluation of prediction metrics and peptide characteristics, we identify key features associated with neoantigen immunogenicity and demonstrate how their integration can refine neoantigen prioritization for future personalized vaccine development.

## Materials and methods

### Data selection

**Figure 1:**
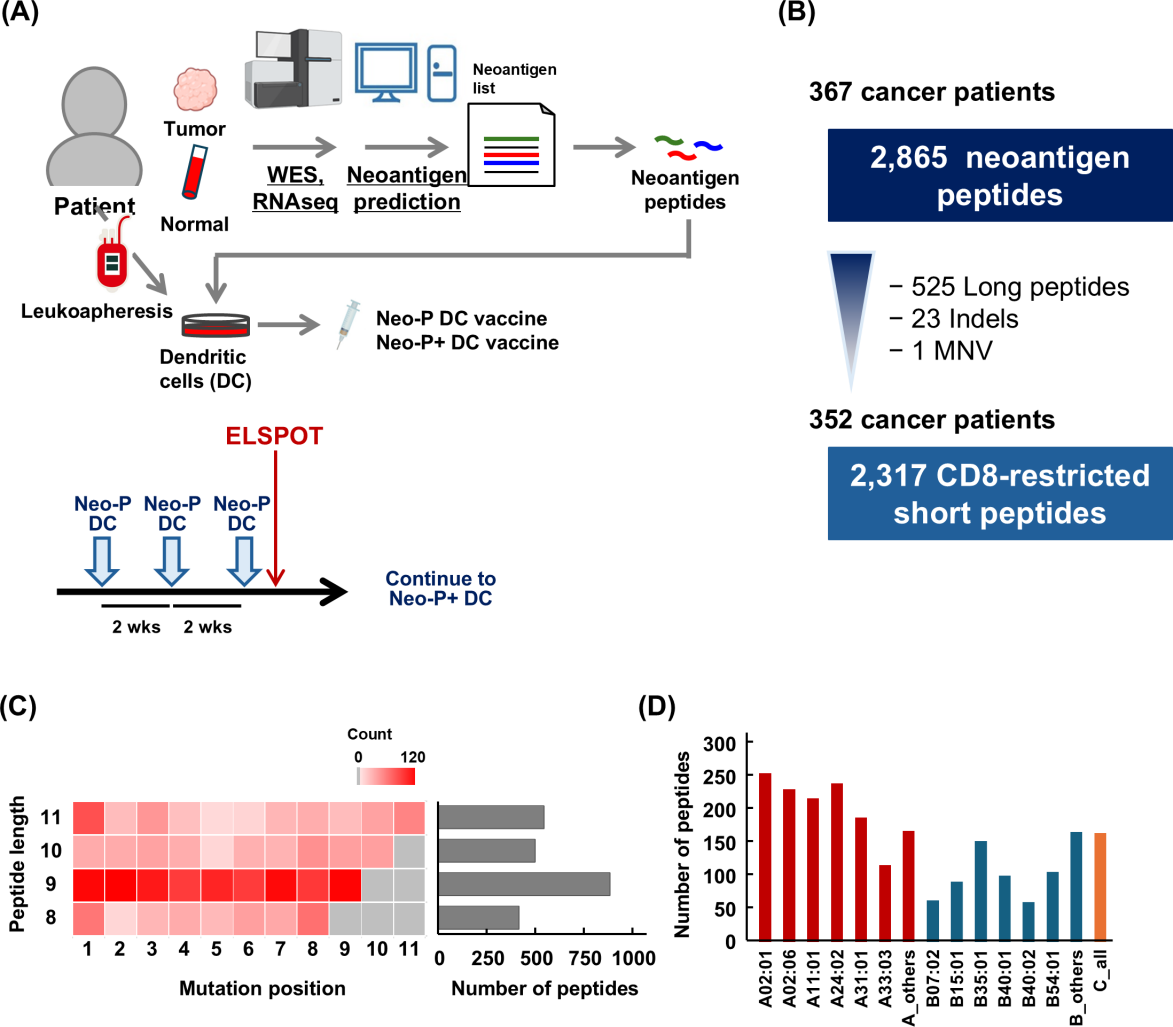
Summary of neoantigen peptides used in personalized neoantigen DC vaccines. **(A)** Workflow of personalized neoantigen peptide-pulsed DC vaccine. Neoantigens were predicted based on whole-exome sequencing (WES) and RNA sequencing (RNAseq) data. Dendritic cells (DCs) differentiated from patient-derived PBMCs were pulsed with synthesized neoantigen peptides to generate neoantigen peptide-pulsed (Neo-P) DC vaccines. After three Neo-P DC vaccinations at 2-week intervals, only neoantigen peptides that elicited IFN-γ ELISPOT responses were subsequently administered as IFN-γ ELISPOT-positive Neo-P (Neo-P+) DC vaccines. **(B)** Summary of peptide dataset used for downstream analyses. A total of 2,865 candidate neoantigen peptides were administered to 367 patients. After filtering to include only single nucleotide variant (SNV)-derived short peptides and excluding those from multi-nucleotide variant (MNV) and insertions/deletions (indels) as well as long peptides, 2,317 peptides administered to 352 patients were retained for analysis. **(C)** Heatmap showing the distribution of peptide length (8–11 amino acids) and mutation position within the peptide. Color intensity represents the number of peptides observed for each length-position combination. The histogram on the right indicates the total peptide count for each peptide length. **(D)** Distribution of predicted neoantigen peptides according to patient HLA class I alleles. The x-axis represents HLA types, and the y-axis indicates the number of peptides. HLA alleles with limited representation (less than a 10% frequency) were grouped as “A_others,” “B_others,” and “C_all.”.

This study aimed to evaluate the characteristics of immunogenic neoantigen peptides using data from cancer patients treated with personalized neoantigen peptide-pulsed DC vaccination. From November 2018 to October 2023, a total of 367 patients, including those reported in our previous studies (**Figure 1A**) (8–14), received personalized neoantigen peptide-pulsed DC therapy at the Department of Cancer Immunotherapy, Fukuoka General Cancer Clinic (Fukuoka, Japan). Across these patients, 2,865 candidate neoantigen peptides (including both short and long peptides designed to induce CD8^+^ and CD4^+^ T cells) were administered. In this analysis, we focused on 2,317 short peptides derived from single nucleotide variants (SNVs) administered to 352 patients, after excluding 12 short insertion-deletion (indel) peptides, one short multi-nucleotide variant (MNV) peptide and 525 long peptides targeting HLA class II molecules (**Figure 1B**). This study complies with the Declaration of Helsinki and was approved by the internal review boards of each institution (FGCC-EC009, B2023-056-01, 2018-GA-1021).

### Neoantigen DC vaccine therapy

Neoantigen DC vaccine therapies were conducted at Fukuoka General Cancer Clinic (Fukuoka, Japan) following the procedures based on the requirements for class III regenerative medicine under the Japanese Act on the Safety of Regenerative Medicine after obtaining written informed consent, as previously described (**Figure 1A**) (8–14).

Neoantigen prediction for personalized neoantigen DC vaccines was performed as previously described (2, 3). Briefly, HLA class I and class II genotypes were determined using OptiType (15) and PHLAT (16). The binding affinities of short peptides with 8- to 11-mer length for HLA-A, HLA-B and HLA-C, and long peptides with the length of 15- to 18-mer for HLA-DRB1 were estimated by NetMHC-3.4/NetMHCpan-2.8 and NetMHCII-2.2/netMHCIIpan-3.1, respectively (2, 3). Neoantigen peptides with IC_50_ less than 500 nM were selected as candidates for personalized vaccine design.

DC vaccines were established as previously described (8–14). Briefly, monocyte-derived immature DCs were prepared from cryopreserved peripheral blood mononuclear cells (PBMCs) obtained by pre-treatment leukapheresis using a plastic adherence method and then matured by adding maturation factors. Short peptides for HLA class I were introduced to mature DCs, while long peptides for HLA class II were introduced to immature DCs. Before use, the neoantigen peptides were confirmed to be negative for endotoxin, β-glucan, and mycoplasma using Toxinometer ET-6000 (Wako Pure Chemical Industries, Ltd., Osaka, Japan) and Mycoplasma detection assay (MycoAlert; Lonza Rockland Inc., Rockland, ME, USA). Peptide-pulsed DCs were suspended in 500 µL saline and administered to the corticomedullary border of the normal inguinal lymph nodes using a 25-G needle under ultrasound guidance (11). The first three vaccinations of neoantigen peptide-pulsed (Neo-P) DCs were performed at 7- to 14-day intervals by ultrasound-guided intranodal injection. The next three vaccinations using IFN-γ ELISPOT-positive Neo-P (Neo-P+) DCs were performed at 3-week intervals. Before and after vaccination, PBMCs were collected to examine the immune response to each neoantigen peptide using IFN-γ ELISPOT assay.

### IFN-γ ELISPOT assay

T cell responses after vaccination were evaluated by an IFN-γ ELISPOT assay using the Human IFN-γ ELISpot Plus Kit (Mabtech Inc., Cincinnati, OH, USA) as previously described (14). Briefly, DCs derived from cryopreserved PBMCs before treatment were pulsed with peptides and were co-cultured with lymphocytes for 48 h. The IFN-γ spots were detected using an Automated ELISpot Reader 0.8 Classic (AID GmbH, Strasberg, Germany). Peptides were classified as “immunogenic” if the IFN-γ spot counts increased a 2.0-fold or greater compared with the control without peptide pulse to confidently capture true immunogenic responses while minimizing false positives due to background noise (7, 17, 18). Peptides not meeting this criterion were classified as “non-immunogenic”.

### Peptide feature analyses

Characteristics of each neoantigen peptide that are potentially significant in the antigen presentation process were calculated as described below.

Hydrophobicity. The overall hydrophobicity of each peptide was assessed using the Grand Average of Hydropathy (GRAVY) score, calculated as the sum of hydropathy values of all amino acids divided by the peptide length (19). Hydrophobicity fraction was calculated as the proportion of hydrophobic residues, including V, I, L, F, M, W and C, within the peptide sequence as reported previously (7).

TCR recognition. Prediction of TCR recognition probability was performed using the BLOSUM62 amino acid similarity matrix to assess the sequence similarity between a neoantigen and the closest matching known T cell epitope from the Immune Epitope Database (20). Based on the previously fitted model (21), the parameters α = 26 and κ = 4.87 were used.

Binding affinity. Binding affinity of a neoantigen peptide to HLA class I was calculated using NetMHC-3.4/NetMHCpan-2.8, NetMHC-4.0/NetMHCpan-4.1, MHCflurry-2.0 or BigMHC with default parameters (22–27).

Binding stability. Binding stability of a neoantigen-HLA complex was estimated as the half-life (h) using NetMHCstabpan-1.0 (28).

Processing. TAP transport efficiency and proteasomal cleavage scores were predicted using NetCTLpan-1.1 (29). MHCflurry-2.0 was also used to predict processing scores.

Presentation. Peptide presentation likelihood was further predicted using BigMHC, mixMHCpred-3.0 and MHCflurry-2.0 (27, 30).

### Metrics used for performance evaluation

To explore the performance of integrated prediction methods, we analyzed individual predictors as well as all combinations. For ensemble models, raw scores were standardized (Z-score) and averaged to form composite scores. The performance of each prediction method was evaluated using a series of standard classification metrics, including accuracy (ACC), balanced accuracy (BACC), sensitivity, specificity, precision, and the area under the receiver operating characteristic (ROC) curve (AUC), all calculated at the optimal cutoff defined by the Youden index. The calculations were based on four parameters: true-positive (TP), true-negative (TN), false-positive (FP), and false-negative (FN). The metrics were defined as follows:


$$ACC=\frac{TP+TN}{\text{TP}+\text{FN}+\text{TN}+\text{FP}}$$

$$Sensitivity=\frac{TP}{\text{TP}+\text{FN}}$$

$$Specificity=\frac{TN}{\text{TN}+\text{FP}}$$

$$Precision=\frac{TP}{\text{TP}+\text{FP}}$$

All metrics were calculated in R, with AUC values obtained from the continuous prediction scores of each method or combination using the *pROC* package. For combinations, the average prediction score of the involved methods was used as the composite predictor prior to metric computation. Five-fold cross-validation was performed to assess model stability. Directionality of predictors was adjusted where appropriate.

### Statistical analysis

Differences in these features between immunogenic and non-immunogenic neoantigens were assessed using linear mixed-effects models (LMMs), treating immunogenicity as a fixed effect and subjects as a random effect. Associations between prediction scores and peptide immunogenicity were assessed using generalized LMMs (GLMMs) with a binomial distribution and logit link, including random intercepts for subjects. The statistical significance of predictors was determined using likelihood ratio tests (LRT), comparing each full model to a corresponding random-intercept-only model. Correlation coefficients were calculated using Spearman’s correlation coefficients. All statistical analyses were conducted in R. Mixed-effects models were fitted using the lme4 and lmerTest packages. Estimated marginal means were calculated using emmeans. A *P* value< 0.05 was considered statistically significant.

## Results

### Overview of clinically tested neoantigen peptides

To comprehensively characterize the landscape of clinically evaluated neoantigen vaccine candidates, we analyzed data from CD8^+^ T cell-restricted 2, 317 short neoantigen peptides derived from SNVs administered to 352 patients as personalized neoantigen peptide-pulsed DC vaccines (**Supplementary Table 1**). The cohort encompassed a broad spectrum of cancer types, most commonly colorectal cancer (*N* = 75), pancreatic cancer (*N* = 48), breast cancer (*N* = 47), gynecologic cancers (*N* = 41), and esophagogastric cancers (*N* = 34) (**Table 1**). The analyzed peptides ranged from 8 to 11 amino acids in length and included all possible mutation positions within the peptide sequence, with heterogeneous representation across length-position combinations (**Figure 1C**). These peptides were predicted to bind a broad spectrum of HLA-A, -B and -C alleles, commonly observed in Japanese populations. (**Figure 1D**, **Supplementary Table 2**).

**Table 1:** Summary of neoantigen peptides in individual cancer types.

Cancer type	Number of patients	Number of immune-positive patients	% of immune-positive patients	P value (patient-based)*	Number of peptides	Number of immunogenic peptides	% of immune-positive peptides	P value (peptide-based)*
Colorectal	75	27	36.0%	0.79	546	77	14.1%	0.87
Pancreas	48	15	31.3%	0.42	291	37	12.7%	0.25
Breast	47	19	40.4%	0.75	281	44	15.7%	0.096
Gynecologic	41	14	34.1%	0.19	289	32	11.1%	**0.0052**
Esophagogastric	34	14	41.2%	0.71	214	32	15.0%	0.51
Liver	22	7	31.8%	0.65	147	10	6.8%	**4.5×10^-4^**
Lung	21	9	42.9%	0.65	136	20	14.7%	0.76
Head/neck	23	9	39.1%	1.0	142	22	15.5%	0.17
Soft tissue	13	4	30.8%	0.80	86	11	12.8%	0.24
Bladder	10	4	40.0%	1.0	69	11	15.9%	0.78
Kidney	10	6	60.0%	0.16	68	12	17.6%	0.63
Prostate	4	1	25.0%	1.0	28	1	3.6%	0.39
Lymphoma	3	2	66.7%	0.56	12	2	16.7%	1.0
Melanoma	1	1	100.0%	0.38	8	2	25.0%	**2.1×10^-5^**
Total	352	132	37.5%	–	2317	313	13.5%	–

*Statistically significant P values (*P* < 0.05) are indicated in bold.

T cell responses to individual peptides were evaluated by IFN-γ ELISPOT assays using PBMCs collected from patients after vaccination. In this study, we defined immunogenic peptides as peptides with a ≥2.0-fold increase in IFN-γ ELISPOT responses, as described in previous reports (7, 17, 18). Among the 352 patients, 132 (37.5%) exhibited immune responses to at least one neoantigen peptide, with broadly comparable response rates across cancer types (**Table 1**, **Supplementary Table 3**). At the peptide level, 313 (13.5%) of the 2,317 peptides induced detectable immune responses, with similar proportions across most tumor types, typically ranging from 10% to 20%, consistent with those reported in previous studies (2–4).

### Peptide characteristics associated with immunogenicity

**Figure 2:**
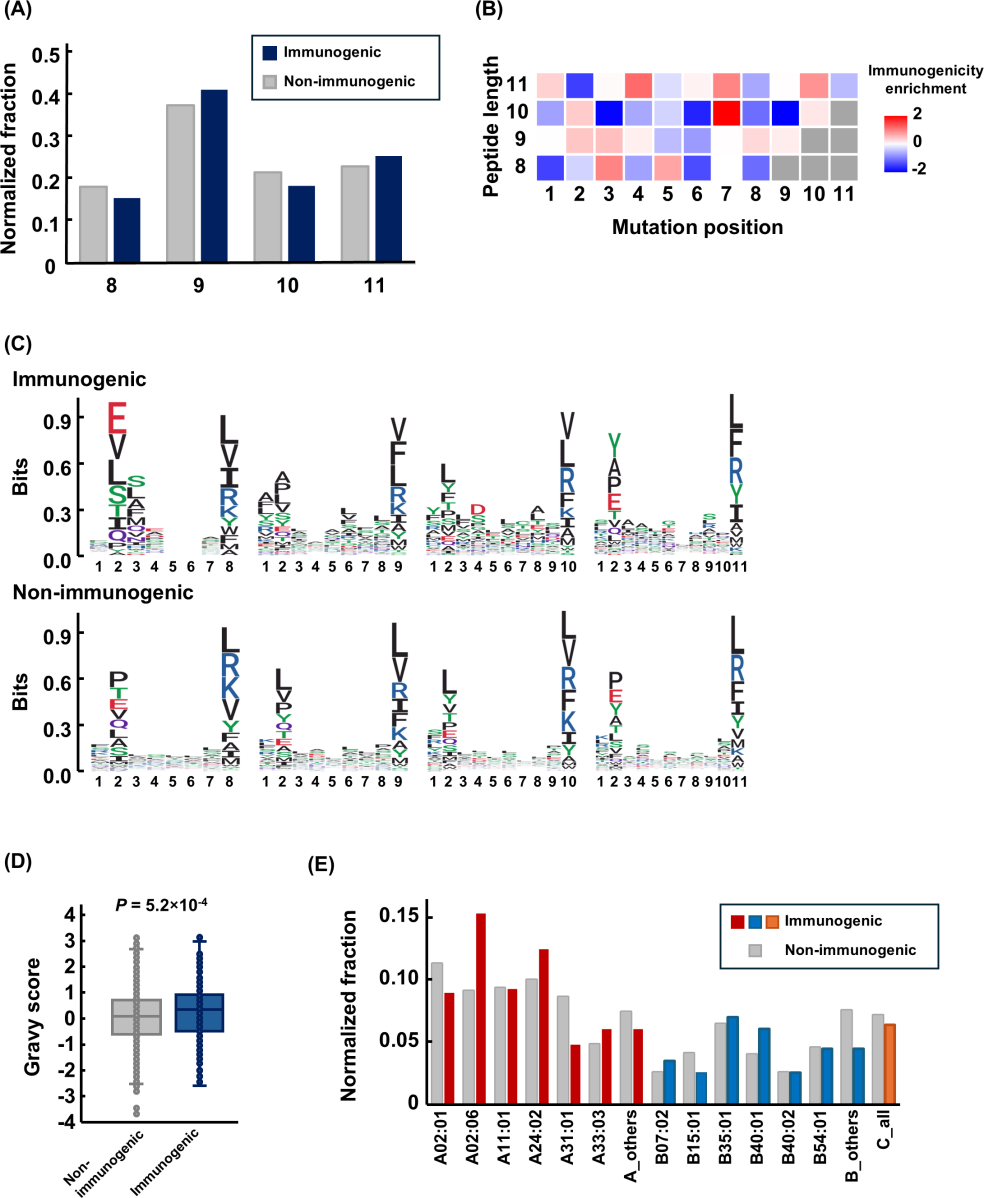
Characteristics of immunogenic neoantigen peptides. **(A)** Distribution of peptide length in immunogenic and non-immunogenic peptides. The x-axis indicates peptide length (8–11 mer), and the y-axis shows the normalized fraction of peptides. **(B)** Heatmap of immunogenicity enrichment according to peptide length and mutation position. The x-axis represents the mutation position within the peptide sequence, and the y-axis shows peptide length (8–11 mer). Color intensity reflects the degree of enrichment of immunogenic peptides relative to non-immunogenic peptides. **(C)** Sequence logos depicting amino acid usage patterns in immunogenic versus non-immunogenic peptides. Sequence logos were generated for 8-mer, 9-mer, 10-mer, and 11-mer peptides using the R package *ggseqlogo*. Each logo illustrates the relative frequency and information content of amino acids at each position in immunogenic (top) and non-immunogenic peptides (bottom). **(D)** Comparison of hydrophobicity (GRAVY score) between immunogenic and non-immunogenic peptides. Boxplots show GRAVY score distributions. **(E)** Distribution of immunogenic peptides across HLA class I alleles. The x-axis indicates HLA types, including grouped categories (“A_others,” “B_others,” and “C_all”) for HLA alleles with fewer than 50 peptides. The y-axis shows normalized peptide fractions.

We first compared the basic characteristics between immunogenic and non-immunogenic peptides. Peptide length showed no apparent association with immune response rates (**Figure 2A**). Similarly, the positions of mutated residues were distributed almost evenly across peptides, indicating that the mutation position alone does not determine immunogenicity (**Figure 2B**). Sequence logo analysis revealed no statistically significant differences in amino acid usage at individual positions between immunogenic and non-immunogenic peptides, while a modest enrichment of alanine at position 1 of 9-mer peptides was observed (OR = 2.4, FDR = 0.094; **Figure 2C**). These results suggest that immunogenicity cannot be explained by individual amino acid substitutions at specific positions, but instead likely reflects the combined effects of multiple peptide features.

Hydrophobicity, quantified as the GRAVY score, differed significantly between the two groups. Immunogenic peptides exhibited higher overall hydrophobicity than non-immunogenic peptides (*P* = 5.2 × 10^-4^; **Figure 2D**). This trend remained consistent when alternative hydrophobicity scoring metrics were applied (7) and when the analysis was restricted to non-anchor residues facing the TCR (*P* = 0.047 and 0.0015, respectively; **Supplementary Figures 1A, B**). In contrast, hydrophobicity at anchor residues was not associated with immunogenicity across the full dataset or among HLA-A-restricted peptides (*P* = 0.25 and 0.40, respectively). However, a significant association was observed among peptides predicted to bind HLA-B and HLA-C molecules (*P* = 0.0072 and 0.00020, respectively; **Supplementary Figure 1C**). No significant association was found for other basic physicochemical features previously reported to influence immunogenicity (**Supplementary Figure 2**).

We further examined immunogenicity across individual HLA alleles and found that peptides predicted to bind HLA-A02:06, A24:02 or B40:01 showed higher proportions of immunogenic peptides, while those for HLA-A31:01 showed a lower immunogenicity rate than others (**Figure 2E**).

### Predicted scores associated with immunogenicity

**Figure 3:**
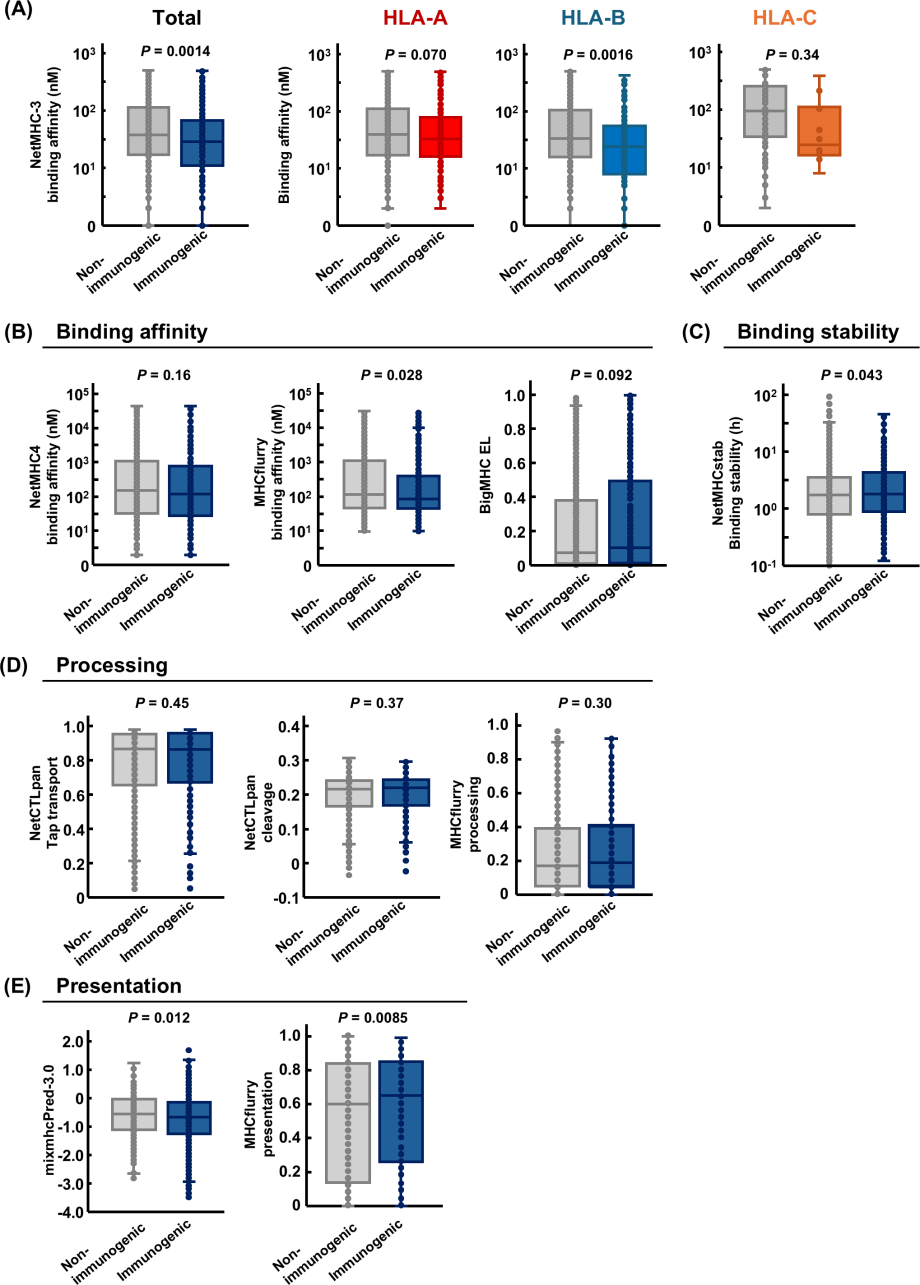
Comparison of predicted scores related to antigen presentation in immunogenic and non-immunogenic peptides. **(A)** Boxplots showing the predicted peptide-HLA binding affinity (IC_50_, nM) of immunogenic versus non-immunogenic peptides. Total HLA-ABC and each HLA class I family (HLA-A, HLA-B and HLA-C) (left to right). **(B–E)** Comparison of predicted scores for antigen presentation-related processes, including binding affinity (NetMHC-4.0, MHCflurry, BigMHC) **(B)**, stability (NetMHCstab) **(C)**, processing (NetCTLpan TAP transport, NetCTLpan cleavage, MHCflurry processing) **(D)**, and presentation (mixMHCpred-3.0, MHCflurry presentation) **(E)**. Each boxplot shows the distribution of scores for immunogenic (blue) and non-immunogenic (gray) peptides.

To further dissect the possible determinants of immunogenicity, we compared multiple prediction scores related to antigen presentation between immunogenic and non-immunogenic peptides. Since antigen presentation involves multiple sequential steps, including proteasomal cleavage, TAP transport, HLA binding and peptide-HLA complex stability, we evaluated representative tools corresponding to each step. We first examined HLA class I binding predictions (**Figure 3A**). NetMHC-3, which is used in our neoantigen vaccine pipeline (8–14), showed that stronger predicted binding affinity was associated with higher immunogenicity (mean IC_50_ values of immunogenic and non-immunogenic peptides: 63 nM vs 83 nM, respectively, *P* = 0.0014). Similar trends were observed for peptides restricted for HLA-A, -B and -C (*P* = 0.070, 0.0016 and 0.34, respectively). When the affinity cutoff was adjusted, a modest increase in the proportion of immunogenic peptides was observed (from 13.5% at <500 nM to 14.7% at <50 nM, *P* = 0.027), indicating that although binding affinity is an important determinant of immunogenicity, adjusting affinity thresholds alone is insufficient to enrich immunogenic peptides (**Supplementary Table 4**).

We next compared alternative binding prediction models (**Figure 3B**). Although NetMHC-3 and its updated version, NetMHC-4, were moderately correlated (Spearman ρ = 0.57, *P* = 3.3 × 10^-54^; **Supplementary Figure 3**), NetMHC-4 showed only a modest association with immunogenicity (*P* = 0.16). Similarly, MHCflurry binding affinity and BigMHC predictions also showed borderline significance (*P* = 0.028 and 0.092, respectively). NetMHCstabpan stability scores, which predict peptide-HLA binding stability, differed significantly between the two groups, with higher predicted stability observed in immunogenic peptides (*P* = 0.043; **Figure 3C**).

Among antigen processing and presentation prediction tools, TAP transport scores and proteasomal cleavage scores from NetCTLpan as well as MHCflurry processing scores showed no significant differences between the two groups (*P* = 0.45, 0.37 and 0.30, respectively; **Figure 3D**). In contrast, presentation scores predicted by MixMHCpred-3.0 and MHCflurry showed significant associations with immunogenicity (*P* = 0.012 and 0.0085, respectively; **Figure 3E**).

### Predictive models for neoantigen immunogenicity

**Figure 4:**
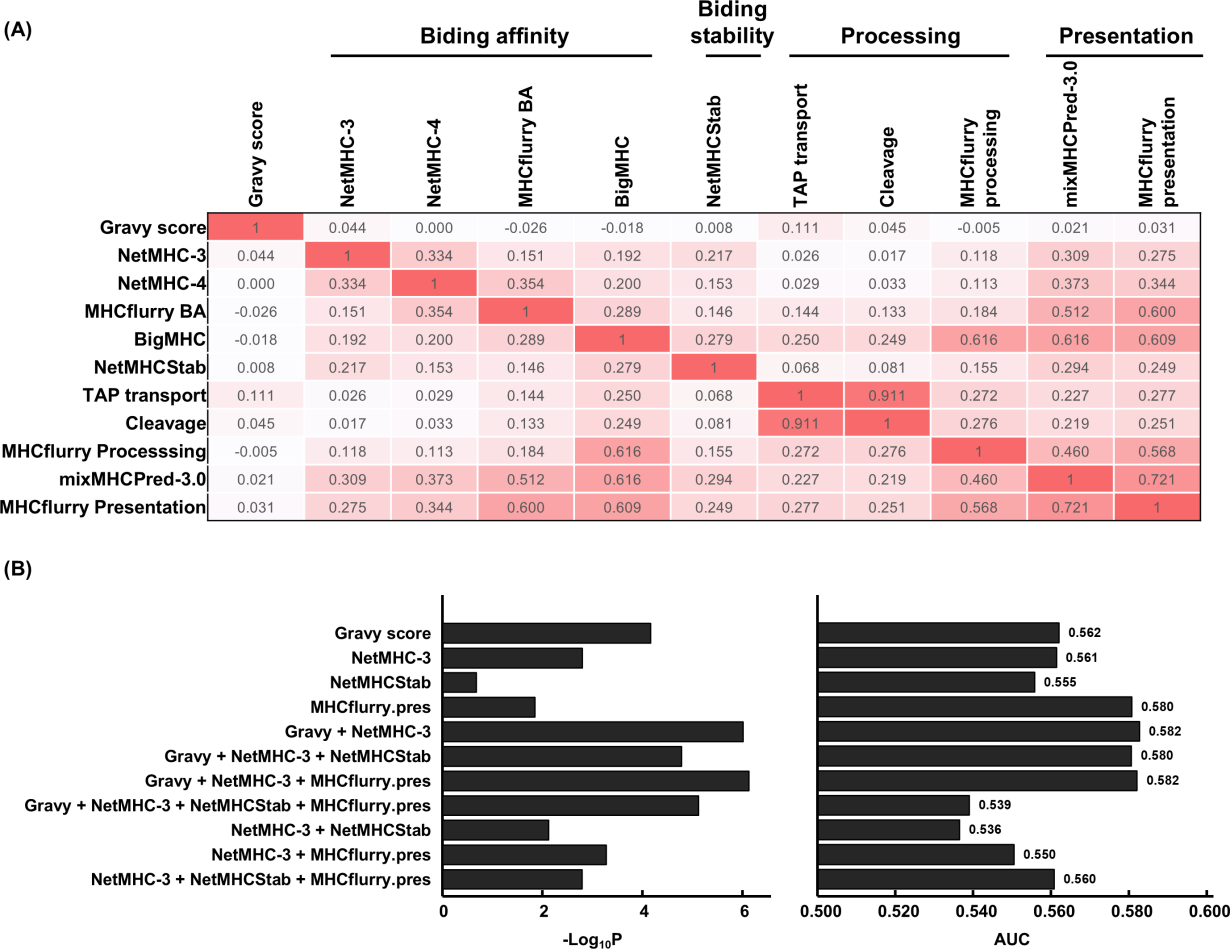
Predictive scoring for neoantigen immunogenicity. **(A)** Heatmap showing pairwise correlations among peptide-intrinsic features (GRAVY score) and representative antigen presentation-related prediction tools, including binding affinity, binding stability, presentation, and processing scores. **(B)** Bar plot showing the statistical association of individual prediction scores and selected composite models with immunogenicity, expressed as -log_10_ (*P* value) from univariate comparisons between immunogenic and non-immunogenic peptides (left). Bar plot comparing performance across selected individual and combined prediction tools (right). Higher AUC values indicate better separation between immunogenic and non-immunogenic peptides.

We further examined pairwise correlations among all immunogenicity-associated scores (**Figure 4A**). As expected, tools modeling similar aspects of antigen presentation showed moderate correlations. In particular, MixMHCpred-3.0 and MHCflurry presentation scores were strongly correlated (Pearson *r* = 0.72), consistent with their shared focus on peptide-HLA presentation. MHCflurry-processing and BigMHC scores also exhibited a moderate correlation (Pearson *r* = 0.62). In contrast, the GRAVY score and antigen-processing-related metrics such as TAP transport and proteasomal cleavage showed weak correlations with most presentation and affinity predictors, indicating that these parameters capture largely independent aspects of peptide biology. Notably, combining these complementary predictors, particularly binding affinity (NetMHC-3), peptide hydrophobicity (GRAVY score), and presentation likelihood (MHCflurry-presentation score), substantially improved separation between immunogenic and non-immunogenic peptides (*P* = 8.0×10^-6^, **Figures 4B** left). Together, these findings support the idea that hydrophobicity and antigen-processing features provide non-redundant information not captured by affinity- or presentation-based tools alone.

Based on these observations, we then evaluated the classification performance of individual and composite models that significantly discriminated immunogenic from non-immunogenic peptides, using ROC analysis (**Figure 4B** right). No single predictor achieved strong discriminative power (AUC range: approximately 0.53-0.56), underscoring the inherent difficulty of predicting neoantigen immunogenicity from sequence-derived features alone. However, composite models integrating physicochemical properties with presentation-oriented scores consistently improved both AUC and balanced accuracy (maximum AUC: 0.582). In particular, combinations incorporating the GRAVY score with affinity- or presentation-based predictors achieved the best overall performance, highlighting the value of multi-feature integration for neoantigen prioritization in personalized vaccines and T cell-based therapies.

## Discussion

In this study, we evaluated the *in vivo* immunogenicity of short (8-11-mer) HLA class I neoantigen peptides in 352 cancer patients treated with personalized neoantigen peptide-pulsed DC therapy. Among 2,317 peptides tested, 13.5% elicited detectable T cell responses meeting the ≥2.0-fold threshold, higher than the ~6% immunogenicity rate reported in large benchmarking efforts such as the TESLA consortium (7, 31, 32). Nevertheless, most peptides remained non-immunogenic, underscoring the persistent challenge of improving neoantigen selection. With over 2,000 peptides analyzed across diverse malignancies, this represents one of the largest clinically annotated neoantigen vaccine cohorts to date (**Table 1**, **Supplementary Table 3**). Although the number of peptides used per patient was limited and pre-selection bias cannot be excluded, and the classification of immunogenic responses may be influenced by the threshold used, the direct *in vivo* assessment after vaccination provides a rare opportunity to link computational prediction with functional immunogenicity.

A consistent peptide-intrinsic feature associated with immunogenicity was increased hydrophobicity. Immunogenic peptides showed higher GRAVY scores, an association that remained significant when analyses were restricted to non-anchor, TCR-facing residues. Similar enrichment of hydrophobic residues has been reported in mass spectrometry-defined immunopeptidomes (33, 34), supporting the biological plausibility of this observation. However, position- and locus-specific analyses revealed complexity. Hydrophobicity at non-anchor positions was significantly associated with immunogenicity in HLA-A-restricted peptides but showed weaker associations for HLA-B and HLA-C-restricted peptides. Sequence logo analysis revealed that HLA-B and HLA-C peptides show lower representation of basic residues and relative enrichment of hydrophobic amino acids at anchor positions compared to HLA-A (**Supplementary Figure 4**). These findings suggest that differences in anchor residue composition across HLA types may contribute to the observed variation in the association between hydrophobicity and immunogenicity. Together, these findings suggest that the impact of hydrophobicity on immunogenicity is context-dependent effects and influenced by HLA restriction.

Integration of NetMHC-based affinity predictions with additional presentation-related and physicochemical features modestly but consistently improved discrimination between immunogenic and non-immunogenic peptides (**Figure 4**). Combinations of binding affinity, presentation likelihood, and hydrophobicity achieved stronger statistical separation than individual predictors, highlighting the value of integrating orthogonal features rather than relying solely on binding affinity. However, the improvement was modest, partly reflecting the fact that candidate neoantigens were pre-selected based on HLA-binding predictions prior to clinical use, resulting in a relatively homogeneous set of peptides in which further discrimination may be more difficult. Future studies incorporating additional features, such as TCR-peptide interactions will be necessary to further improve predictive performance and assess the generalizability of these findings.

We note several limitations in this study. First, although CD4^+^ T cell responses play a critical role in antitumor immunity and have been prominent in several neoantigen vaccine trials (2–4, 12–14), our analysis was restricted to CD8^+^ T-cell-restricted short peptides for several reasons. The number of long peptides in the present dataset was limited. In addition, in our ELISPOT assays using autologous DCs as stimulators, we were not able to distinguish whether the observed responses were mediated specifically by CD8^+^ or CD4^+^ T cells, nor to identify the core peptide sequences presented by HLA molecules. Furthermore, reliable HLA class II prediction remains less established compared to HLA class I. Therefore, further studies specifically addressing CD4^+^ T cell responses and long peptides will be important.

In addition, neoantigen immunogenicity is patient-specific: non-immunogenic peptides in one individual may elicit responses in others due to differences in HLA background, immune status, and TCR repertoire (1, 35). The cohort analyzed in this study reflects a real-world clinical setting and includes heterogeneous patient backgrounds that were not fully controlled for. Therefore, the absence of a detectable immune response does not necessarily indicate that a peptide lacks immunogenic potential. While these limitations could not be addressed within the current dataset, they represent important considerations for future studies with more balanced and controlled cohorts.

Finally, measurable T cell responses do not necessarily translate into antitumor activity, which also depends on antigen expression levels, antigen processing and presentation, and the immune-active or suppressive tumor microenvironment (36, 37). Incorporating tumor-intrinsic factors such as mutant RNA expression, surface neoantigen–HLA complexes, and immune evasion pathways will be essential for developing more clinically relevant prediction frameworks (7, 38).

Overall, by linking computational predictors with empirical immunogenicity data from clinically tested neoantigens, this study provides a practical framework for improving neoantigen prioritization in precision cancer immunotherapy.

## Data Availability

The original contributions presented in the study are included in the article and its **Supplementary Material**. Further inquiries can be directed to the corresponding author.
