# Supplementary material for: Profiling immunogenic neoantigen peptides elicited by personalized neoantigen vaccine in cancer patients
Source: Front Immunol. 2026 Jun 9;17:1829509. doi: 10.3389/fimmu.2026.1829509 (PMC13286890; doi:10.3389/fimmu.2026.1829509)
Supplement: Supplementary file 1 [file DataSheet1.pdf]

Supplementary Material for

Profiling immunogenic neoantigen peptides elicited by personalized neoantigen vaccine in cancer patients

Peng Zhao, Clara Effenberger, Saki Matsumoto, Takafumi Morisaki, Yu Ishii, Masayo Umebayashi, Hiroto Tanaka, Norihiro Koya, Shinichiro Nakagawa, Kenta Tsujimura, Yusuke Nakamura, Takashi Morisaki, Kazuma Kiyotani*

*Corresponding author: Email: k-kiyotani@nibn.go.jp.

Index of Supplementary Data

Supplementary Fig. 1. Hydrophobicity characteristics of immunogenic and non-immunogenic neoantigen peptides.

Supplementary Fig. 2. Peptide-intrinsic features and predicted TCR recognition of immunogenic and non-immunogenic neoantigen peptides.

Supplementary Fig. 3. Correlation between NetMHC binding affinity predictions across different versions.

Supplementary Figure 4. Sequence logos depicting amino acid usage patterns in immunogenic versus non-immunogenic peptides stratified by HLA type.

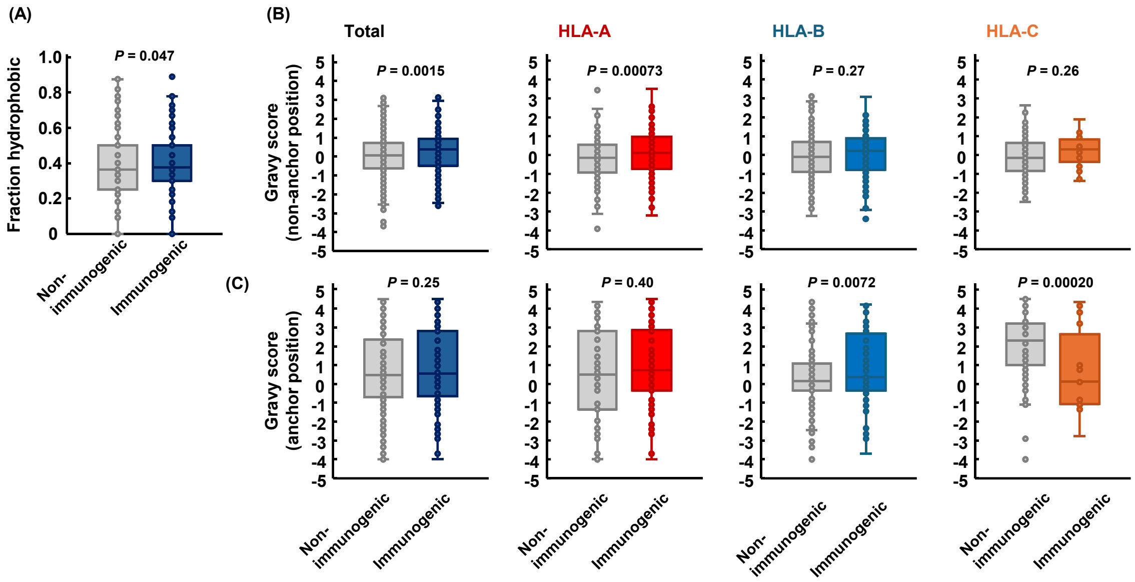

Supplementary Figure 1. Hydrophobicity characteristics of immunogenic and non-immunogenic neoantigen peptides.

(A) Comparison of peptide hydrophobicity between immunogenic and non-immunogenic neoantigen peptides, calculated using the fraction hydrophobicity score (7). **(B-C)** GRAVY score distributions for non-anchor residues (**B**) and anchor residues (**C**) across total HLA-ABC peptides and peptides restricted to HLA-A, HLA-B, and HLA-C.

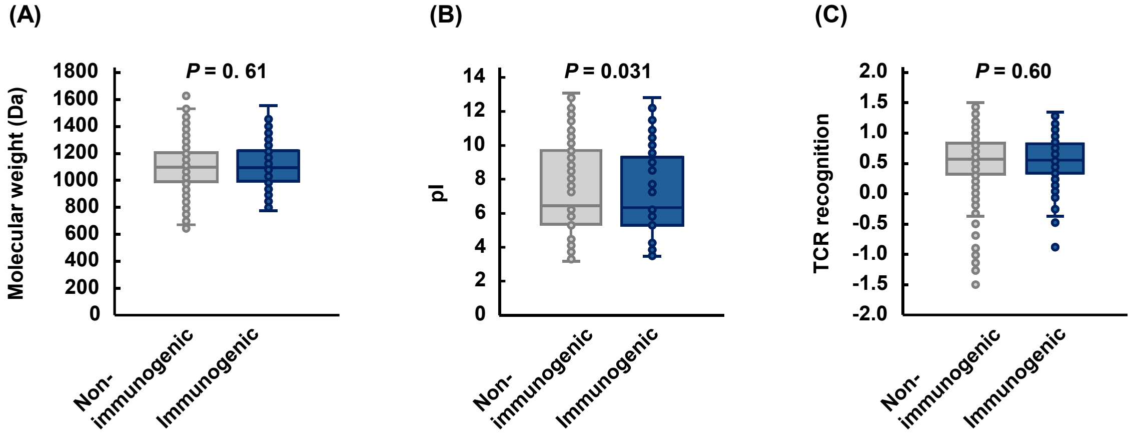

Supplementary Figure 2. Peptide-intrinsic and TCR recognition–related features of immunogenic and non-immunogenic neoantigen peptides.

(A-C) Comparison of peptide-intrinsic features and predicted TCR recognition probability between immunogenic and non-immunogenic neoantigen peptides, including molecular weight **(A)**, isoelectric point (pI) **(B)**, and predicted TCR recognition probability **(C)**.

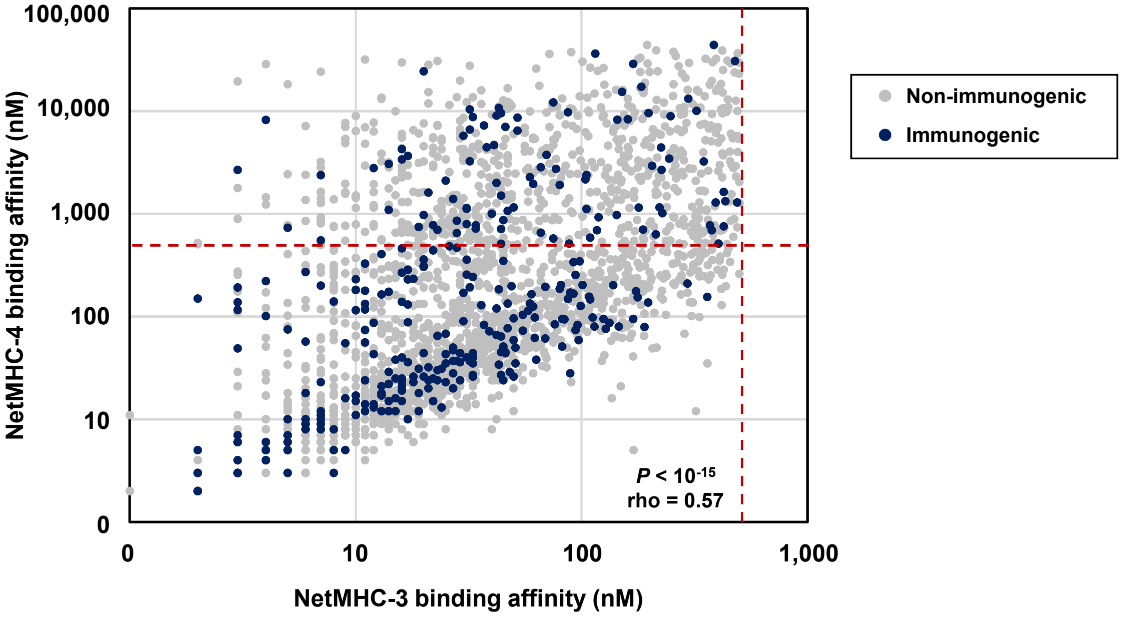

Supplementary Figure 3. Correlation between NetMHC binding affinity predictions across different versions.

Correlation of predicted peptide-HLA binding affinity (nM) between NetMHC-3 and NetMHC-4 for neoantigen peptides. Immunogenic peptides are shown in navy, and non-immunogenic peptides are shown in gray.

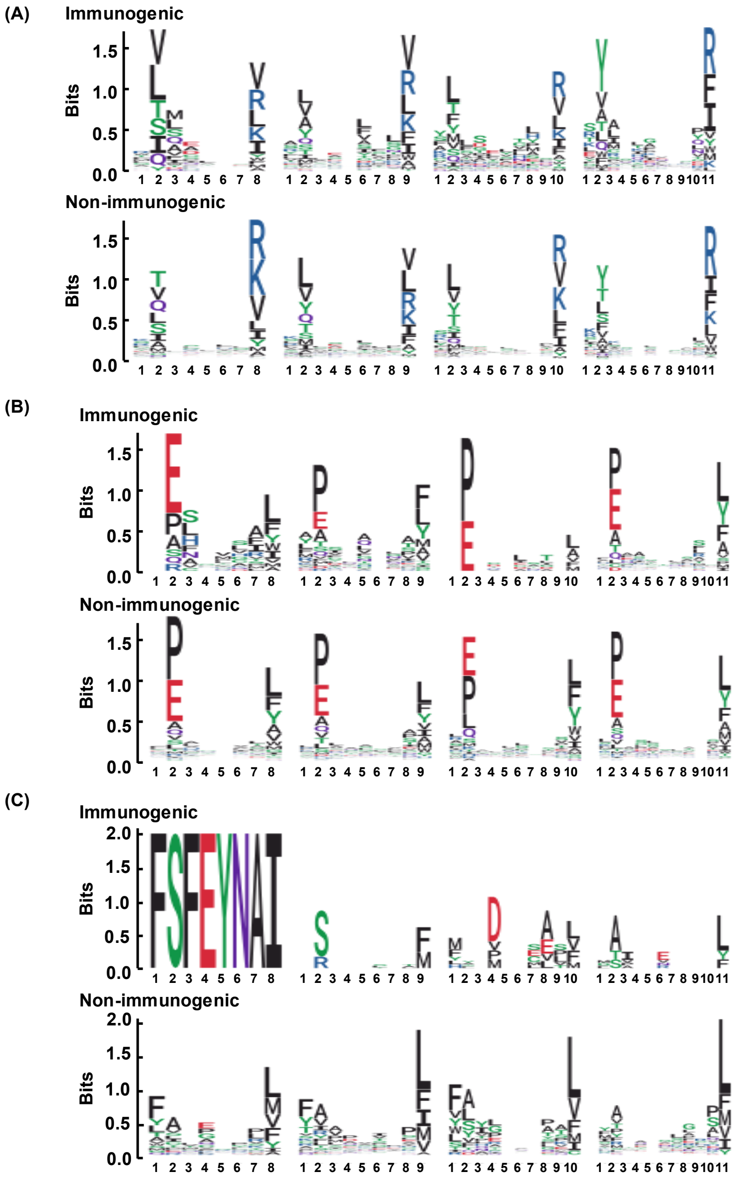

Supplementary Figure 4. Sequence logos depicting amino acid usage patterns in immunogenic versus non-immunogenic peptides stratified by HLA type. (A-C) Sequence logos were generated for 8-11-mer peptides using the R package ggseqlogo. Immunogenic peptides (top) and non-immunogenic peptides (bottom) are shown separately for HLA-A (**A**), HLA-B (**B**) and HLA-C (**C**). Letter height represents amino acid frequency and information content, while colors indicate physicochemical properties (acidic residues in red, basic residues in blue, hydrophobic residues in black, neutral residues in purple, polar residues in green).
